# Smart Insole Based on Flexi Force and Flex Sensor for Monitoring Different Body Postures

**DOI:** 10.3390/s22155469

**Published:** 2022-07-22

**Authors:** Rafique Ahmed Lakho, Zamir Ahmed Abro, Jun Chen, Rui Min

**Affiliations:** 1 Center for Cognition and Neuroergonomics, State Key Laboratory of Cognitive Neuroscience and Learning, Beijing Normal University, Zhuhai 519087, China; rafiqueahmed116@hotmail.com (R.A.L.); 202011059015@mail.bnu.edu.cn (J.C.); 2 CNR IPCF, Bari Division c/o Dipartimento di Chimica, Universita di Bari, Via Orabona 4, I-70126 Bari, Italy; 3 Department of Textile Engineering, BUITEMS, Airport Road, Quetta 87300, Pakistan; zamirabro@hotmail.com

**Keywords:** smart insoles, Flexi force sensor, flex sensor, plantar pressure monitoring, body posture

## Abstract

This study aims to fabricate smart insoles using wireless Flexi force and bend sensing technology. Polyvinyl chloride (PVC) film was chosen as the substrate to hold all the sensors. The developed smart insole has a three-layer structure (insole-PVC layer-fabric layer) and is calibrated in an isolation laboratory to evaluate its measurement performance. One male volunteer subject exhibited four different body postures, namely tree pose, forward-leaning, squatting, and forward folding pose. Changes in pressure distribution were considered to be similar for the forward, squat, and forward-folded positions. When subjects performed a full squat, the flex sensor exhibited maximum flexion during the squat position, and the flex sensor response against the squat pose was found to be higher by about 18.18% than in the forward lean, respectively. The tree pose has the highest error rate at the first metatarsal, about 18.6%, of which the maximum absolute relative error of the sensor is less than 5%. Plantar pressure distribution and body posture measurements were successfully validated using Flexi force and flex sensors embedded in the smart insole. The smart insole proposed in this research work has broader prospects for clinical application.

## 1. Introduction

The study of plantar pressure measurement has a vibrant role in the area of foot health monitoring. In various parts of the globe, patients have to travel to clinics frequently due to a lack of available medical expertise [1,2]. Analysis of plantar pressure distribution is an important part of clinical gait examination in rehabilitation medicine [3,4,5], which is the basis for measuring abnormal foot pressure [6,7] and evaluating peripheral neuropathies, diabetes mellitus, neuropathic ulcers, and musculoskeletal disorders. Abnormal foot pressure may cause injuries at different positions of the foot and can lead to foot ulceration. In diabetic patients, foot ulcers lead to a majority (85%) of lower-extremity amputations [8,9]. Andrew et al. [10] studied that neuropathic diabetic patients with foot ulceration have abnormally high pressure under the feet during walking. The significant applications of plantar pressure measurement include analyzing different body postures [11], sports biomechanics [12], biomedical especially diagnosis of foot-related diseases [13], designing footwear in the shoe industry [14], athlete performances [15], several sports activities [16], observing dynamics of obese people, monitoring posture allocation and human’s daily life activities such as standing, walking, jogging and running [17,18,19].

There are multiple thin-film pressure sensors commercially available in the market, such as the Quantum Tunneling Composite (QTC) sensor (Peratech Ltd., Richmond, North Yorkshire, UK) and Force Sensitive Resistors (FSR) sensor (Interlink Electronics, Camarillo, CA, USA), but some unique properties of the Flexi force sensor (Tekscan, Boston, MA, USA) are more advantageous than other sensors [20,21]. Flexi force sensors have less linearity error, repeatability of ±2.5% of full scale, hysteresis and drift <5% of full scale [22], minor temperature effects of less than 0.36% per degree Celsius and <5 ms response time. *C. Lebosse* et al. 2008 [23] utilized the Interlink FSR and Tekscan Flexi force sensors in robotic and biomechanical applications. The linearity-based results achieved from the Flexi force sensor were better than the Interlink sensor. They concluded that both the sensors could work efficiently in the high magnetic field. *F. Vecchi* and their colleagues [24] have accomplished a series of measurements for evaluating both Tekscan Flexi force and Interlink FSR sensors. The results showed that the Flexi force sensor responds much better in terms of repeatability, linearity, time drift, and dynamic accuracy. Flexi force sensors have been mainly used in biomedical applications to measure interfacial pressure such as orthotic and prosthetic assistive devices [25,26], crutches, braces, force exerting medical devices such as laparoscopic [27], pressure in compression garments [28], the pressure under a digital tourniquet [29], forces in helmet [30] and gait analysis devices [31,32].

The most common form of strain sensor is the flex sensor, which can measure the bending of human body joints or devices. Flex sensors are cheaper and easy to assemble as compared to strain sensors [32]. Optical sensors are categorized as a type of flex sensor in which bending changes the refractive index of the fiber optic cable [33]. Due to some remarkable properties of the flex sensor, such as low price, robustness, and long life, it demonstrates nonlinear response and minor sensitivity at small bending angles [34]. Flex sensors are widely used as goniometers in biomedical devices for analyzing human body postures [35,36,37], such as flexion of the wrist, elbow and knee [38], and in human–machine interface (HMI) mechanisms to regulate their operation using gestures. Flex sensors are also used in gloves [39] to recognize sign language and health monitoring systems to measure the angle and motion of muscle joints [40]. Recently, flex sensors have been used in wearable technology for conducting real-time monitoring in the everyday environment [41,42,43,44,45,46,47,48,49].

Despite the extensive use of the Flexi force sensor and flex sensor in various applications involving body/device interfaces, there is limited data available for using both sensors together in any form. Previous studies only focused on the real-time gait analysis of different subjects during walking. The human body performs several body positions every day; therefore, it is necessary to analyze the distribution of plantar pressure in different positions. In this study, the smart insoles were calibrated in an isolated laboratory. Smart insoles are based on four Flexi force sensors, and a flex sensor was designed to monitor changes in plantar pressure while flexing the foot in different body positions in a static position. One male volunteer subject performed all postural measurements by varying different body positions. We combined the monitored pressure distribution and bending state data to analyze changes in body posture. Finally, the relative changes in the center of gravity were investigated. These smart insoles can be used to sense various body postures in static and motion positions.

## 2. Smart Insole Development

### 2.1. Flexi Force Sensor

The Flexi force sensor is an ultra-thin flexible printed circuit with paper-thin dimensions and high flexibility, so the sensor can be easily integrated into any structure [50]. Flexi force sensors consist of a two-layer (polyester/polyamide) film. A conductive material (silver) is deposited on each layer, followed by a layer of pressure-sensitive ink 8. When a force is applied to the effective sensing area of the sensor, it causes the resistance of the sensing element to change, i.e., inversely proportional to the applied force. When no force is applied, the resistance of the sensing area is high (over 5 M-ohms). However, the resistance of the Flexi force sensor decreases as the force applied to the sensing unit increases, and the control circuit converts the change in resistance into a linear voltage. Suitable for such applications, this sensor can measure force without disturbing any test dynamics. In this study, the A201 model of the Flexi force transducer (0–100 lbs.) was used.

### 2.2. Flex Sensor

The resistive flex sensor is a typical sensor that can measure various bending statuses and the amount of deflection [33]. Flex sensors are broadly used in several applications, such as biomedical devices to record static and dynamic postures, virtual reality gaming consoles, automotive controls, robotics [38,39], fitness products, animatronics, and analyzing various body postures [40]. Despite the remarkable properties of flex sensors, such as low price, long life, and robustness, it often validates the nonlinear response and inferior sensitivity at small bending angles. The commercial flex sensor contains a flexible plastic substrate on which a resistive layer and carbon/polymer ink are printed. Polyester or polyimide coatings are often applied to protect the resistive layer of the flex sensor from external environmental factors [31]. The dimensions of the sensing area of the flex sensor are 110 mm × 40 mm × 0.18 mm (length × width × thickness) and have two pins at the end; one is a power pin (printed with polymer ink), and another is the ground. The flex sensor is categorized as a resistor whose resistance changes at various bend angles. The flex sensor transforms the bend into electrical resistance since the resistance is directly proportional to the amount of bend. The greater the bend, the greater the resistance value. When the sensor is in a straight position (unbend), the resistance returns to the initial value.

### 2.3. Smart Insole and Monitoring System

**Figure 1:**
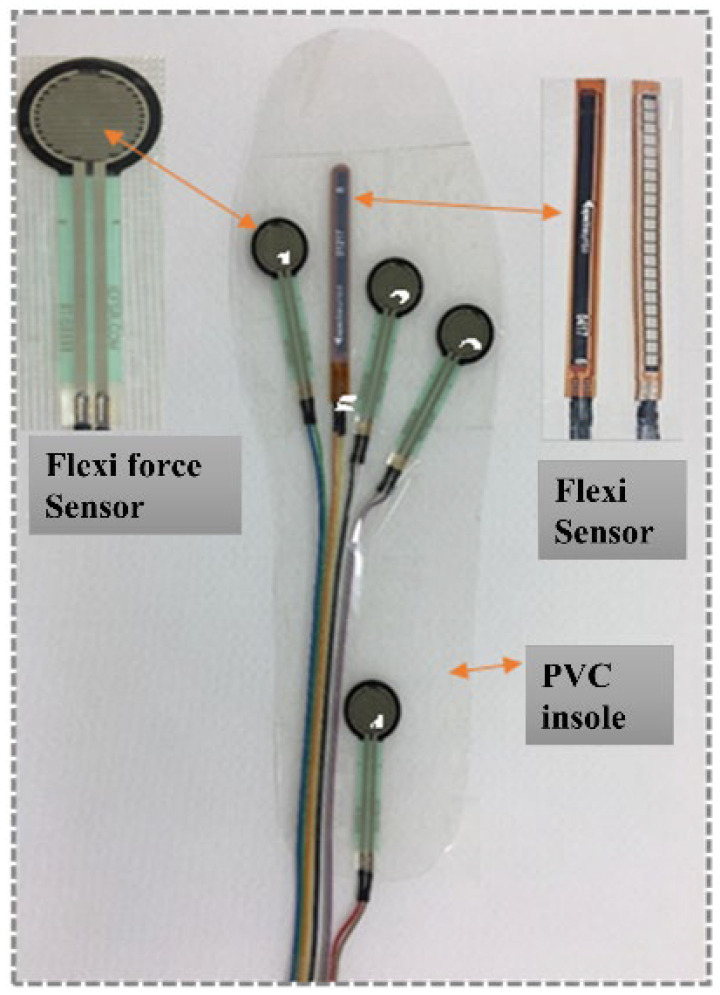
Asymmetric diagram of the encapsulation of the Flexi force and flex sensor on a PVC-based insole.

**Figure 2:**
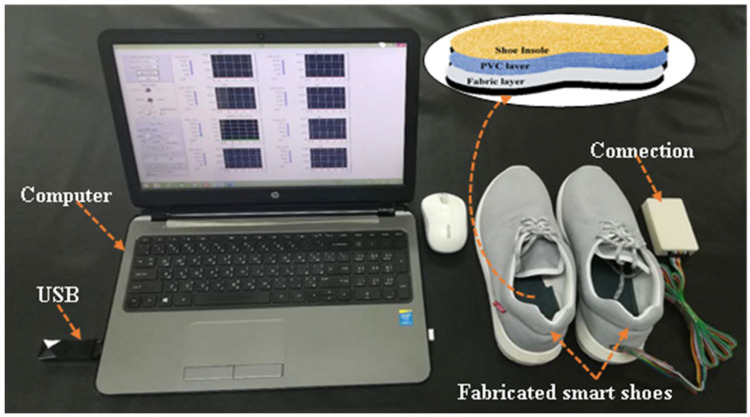
A complete plantar pressure monitoring system for analyzing body postures.

A Polyvinyl Chloride (PVC) sheet was chosen as a base plate for placing the Flexi force sensors and flex sensor. Typical specifications and properties of the Flexi force sensor, flex sensor, PVC material, and men’s sports shoes are given in Table 1. The main purpose for choosing PVC (sheet) material was its properties of lightweight, low thickness, wearer comfortability, smooth surface, and easy sheeting of Flexi force sensors on the surface of PVC. Men’s sports shoe size 42 was selected, and the right (foot) shoe was used for experiments. Primarily, the insole of the right shoe was taken out, and a PVC sheet was cut by using scissors with the same dimensions as the insole of the right shoe. As the maximum pressure could be sustained on the insole when the man was in a standing position, four Flexi force sensors were selected and placed on the PVC insole [41]. The 1st, 2nd, 3rd, and 4th Flexi force sensors were, respectively, placed on the first metatarsal position, the fifth metatarsal position, the third metatarsal position, and the heel position of the insole. The sensors were bonded to their specific positions by using simple scotch tape over the PVC insoles. A Teflon Transparent Adhesive Packaging Tape (3 inch × 65 m) roll with a thickness of 3.5 (mil) was used to fix all the sensors on PVC-based insole, and this PVC insole was further combined with a fabric layer so the sensors could be stable during testing. The Flexi force sensors determined the plantar pressure of the foot at a specific point (1st, 5th, 3rd metatarsus, and heel position) when the body changes posture. Figure 1 shows the positions of four Flexi force sensors on the PVC-based insole. In this study, the flex sensor determined the change in the bend angle of the right foot of the subject while performing different body postures. The position of the flex sensor was chosen between the 1st and 3rd metatarsus of the foot because, at this location, when the subject changes body posture, the maximum bend of the foot can be analyzed. Figure 1 shows the position of the flex sensor on the PVC-based insole. The fabricated insole consists of three layers; first, the actual shoe insole; second, the PVC insole containing both Flexi force sensors and flex sensor; and the third layer was the fabric layer to protect the PVC from both sides (upper layer and lower layer). The arrangement of these three layers is also shown in Figure 2. Finally, all these three layers were combined and stitched around the edges of the insole. The thickness of the final fabricated insole was 4 mm. All the connection wires of Flexi force sensors and flex sensors were taken out at the end (heel position) of the shoe using a small hole. The complete monitoring system is shown in Figure 2, which includes a pair of shoes, the fabricated insole for the right foot, a computer, a rechargeable connection panel, and USB-containing software, which includes a voltage divider to collect the output data, and a Bluetooth device to connect with a laptop and to run the whole system. All the output data were transferred wirelessly from the connection panel to the computer.

### 2.4. Metrological Characterization

The performance of metrological characterization was completed to verify the functionality of the Flexi force and flex sensors. The metrological characterization was performed at room temperature. Four Flexi force sensors and one flex sensor were calibrated and were mounted at the 1st, 5th, 3rd metatarsus, and heel position of the PVC-based insole. Each sensor was calibrated separately since the placement of every sensor was different on the PVC-based insole. It was difficult to apply a constant force on each sensor at the same time, so it was achieved by using five equal weights (length 7.5 cm, width 3.8 cm) to load the sensors. A complete fabricated insole with three layers stitched was used for metrological characterization. The manufactured insole was placed on a smooth surface, and it is stated for 10 s. At the same time, the sensor does not carry any weight. After 10 s, a weight of 5 kg was applied manually to the Flexi force sensor as sensor performance can be seen before fixing the insole inside the shoe. The same procedure was repeated five times every 10 s intervals to simulate the loading process. The total time taken for the loading process was recorded as 50 s. After the loading process was completed, the unloading process was continued by unloading every 10 s.

**Figure 3:**
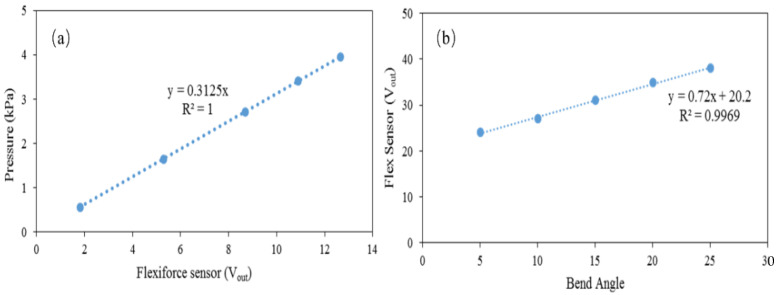
(**a**) A typical relationship between the Flexi force signal change and pressure in the metrological characterization. (**b**) Flex sensor response against change in the bend angle.

Figure 3a illustrates the average relationship between the output of the Flexi force sensor and the upward pressure of all embedded sensors for the designed smart insole. The Flexi force sensor has a linear relationship between the output of the Flexi force sensor and the applied pressure. The output data of the Flexi force sensor continues to increase with the increase in pressure. At the maximum pressure of 4 kPa, the output of the Flexi force sensor was 12.6 V_out_. The flex sensor was also calibrated before inserting it into the smart insole. A piece of paper showing a 180° angle is attached to the table. Initially, the bend sensor was placed at a 0° angle, and after every 10 s, the sensor was moved ahead by 5°. Figure 3b shows the linear correlation between the flex sensor output and the change in the bend angle during a metrological test, and the bend angle is calculated from the output data of Flexi force sensors. In this linear relationship, when the bend sensor is bent at 25°, the bend sensor output reaches approximately 38 V_out_, which indicates that the resistance of the bend sensor increases with the bend angle. The metrological results prove that the bending sensor can be used to determine the bending angle under different body postures.

## 3. Sensors’ Postural Monitoring Methods and Experimental Results

To test the sensing performance of the smart insole, a male volunteer subject was selected to measure the plantar pressure. The selected subject was 24 years old, and he was 1.6 m tall. The subject’s initial weight was 54 kg, and his BMI was 26.3. The right shoe was chosen to insert the smart insole. The subject felt comfortable wearing socks during body pose performances. The connection panel was fixed on the right leg of the subject by using scotch tape. The subject was educated and instructed to maintain as much balance as possible so that the body’s weight could be distributed evenly on the left and right feet. All monitoring tests were carried out at room temperature (25 ± 2 °C). In the study, subjects were asked to form four different poses while using the smart shoes. The poses selected are frequently used by humans in daily life, and detailed descriptions are given below.

**Figure 4:**
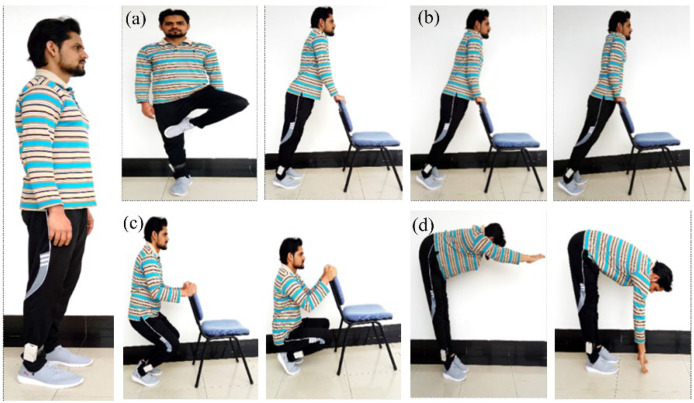
Classification of four different body postures performed by a male subject. (**a**) Tree posture; (**b**) leaning forward posture; (**c**) squat posture; (**d**) forward fold posture. The numbers represent the particular step of the posture.

*First task ‘‘Tree posture’’:* In this test, the subject wore smart shoes and stood upright for 20 s. After 20 s, the subject made a tree pose by lifting the left foot and placing the left foot on the thigh of the right leg, as shown in Figure 4a. The subject maintained this tree position for 20 s, and then returned to an upright position for 20 s. The subject performed the same exercise three times and recorded the output force. The reason for choosing shorter durations in all poses is so that subjects can perform all body poses with ease.

*Second task ‘‘Leaning forward’’:* The subject completed this posture in such a way that only the upper part of the body (from head to hip) moved forward, while the lower part of the body (legs and feet) remained straight. The subject puts his hands on the chair and exerts insufficient force to maintain body balance, as shown in Figure 4b, which does not affect the measurement. In this test, a chart was displayed on the wall, recording the change in posture per centimeter; the subject stood upright close to the wall to perform the forward lean task. Initially, the body is in an upright position for 10 s (first step). The subject was asked to stand up straight in such a way that they felt the greatest pressure in the heel area. The subject’s body moved 9 cm forward and stopped for 10 s (step 2). After 10 s, the body is projected forward again, and the same process is carried out in the third and fourth steps. The total time to complete this pose was 40 s and was controlled by a stopwatch.

*Third task ‘‘Squat position’’:* In this posture, the subject stood up straight for 10 s wearing the smart insole-based shoes. In the second step, the subject was sitting in a chair for 10 s. In the third and final step, the subject completed a full squat by bending their feet for 10 s, as shown in Figure 4c. For this posture, the total time recorded 30 s for the lean forward and chair sitting. The main purpose of using a chair is to stabilize the weight of the subject and minimize errors during the test.

*The fourth task ‘‘Forward fold’’:* This task consists of three steps, and the duration of all steps was kept at 10 s. In the first step, the subject stood up straight, the same as in all previous tasks. In the second step, the subject’s upper body was folded forward at the waist until it reached a 90° angle, as shown in Figure 4d. During this step, the subject’s hand remained straight and pointed downward. In the third and final step, the subject folds forward further until the subject’s hand touches the ground. Similar to the others, this pose takes 30 s to complete. Each test was repeated 5 times to ensure the consistency of the results. The output of the Flexi force and flex sensors were simultaneously displayed on the computer screen. To distinguish the results of the Flexi force and bending sensors, each sensor was marked with a number.

## 4. Postural Assessment Using the Smart Insole

**Figure 5:**
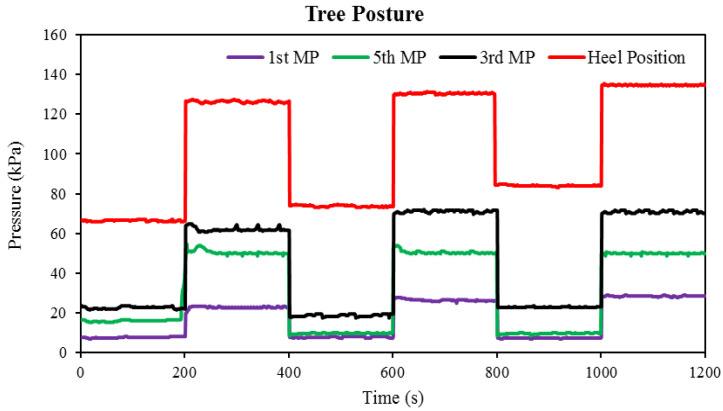
The pressure changes in Flexi force sensors against time during the tree posture.

In order to better understand the posture change phenomenon, the output force value obtained during the posture change process is converted into pressure. The conversion is performed by dividing the force value by the surface area of the insole. Figure 5 illustrates the change in the plantar pressure of all Flexi force sensors with the elapsed time during the tree posture (the first task). The four Flexi force sensors attached to the first, fifth, and third metatarsal and heel positions are characterized by the initial pressure values of the subject when the subject is upright, which are 7.66, 16.129, 22.93, and 66.53 kPa, respectively. All Flexi force sensors exhibit quick changes in pressure, indicating that the recorded data correspond to changes in the subject’s vertical pressure. At the first, fifth, third, and heel positions, the Flexi force sensors showed a constant increase in pressure when the subject changed the pose from a straight point to the tree pose. The body weight of the subject was distributed evenly on both feet while in the standing position, but when the subject converted to the tree pose, the entire body weight was redistributed onto the right foot.

The Flexi force sensor pressure changes depending on applied stress. The maximum recorded value of the plantar pressure in the heel area is 130 kPa, and the position of the first metatarsal is 25 kPa (minimum). The recorded pressure measurements of the fifth and third metatarsus lie between the recorded values of the first metatarsus and the heel position. As this task was performed three times continuously, approximately similar results were achieved, which are shown in Figure 5. This tree posture shows that all the existing Flexi force sensors in smart shoes are reliable and accurate for plantar pressure measurement.

**Figure 6:**
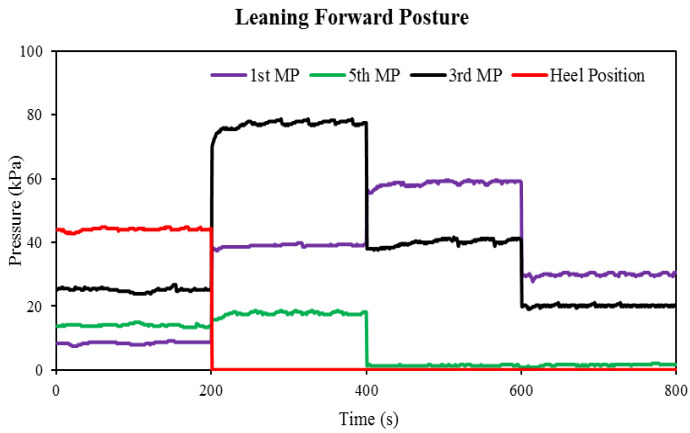
During the leaning forward, the posture pressure changes of Flexi force sensors over time.

**Figure 7:**
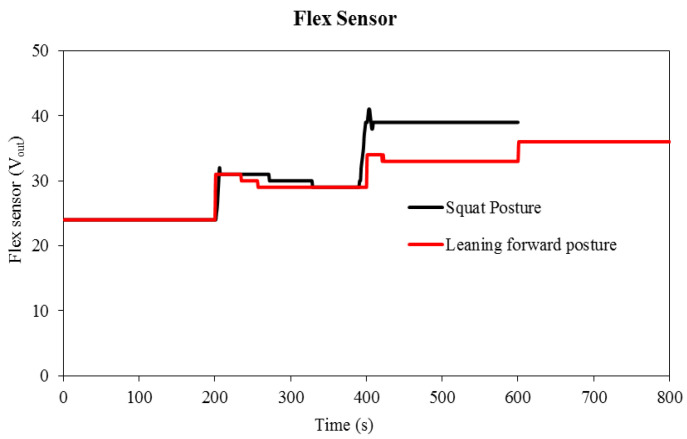
Flex sensor response against time during leaning forward and squat postures.

Figure 6 shows the change in plantar pressure over time when the subject performs the forward-leaning posture (the second task). The values of pressure change were increased at the first metatarsus as the subject started to move forward (2nd step). Conversely, when the subject stopped exercising for 20 s (200–400 s), the pressure stabilized at 39.70 kPa. In the third step, as the body moved further forward, the pressure increased. The highest pressure recorded at the first metatarsus position was also at this step, which was around 59.27 kPa. At the final level, the pressure reduced as compared to the previous steps (2nd and 3rd). The Flexi force sensor assigned to the fifth metatarsal showed a small increase in pressure compared to the initial pressure. As the subject moved for the leaning forward posture of the second step, the contact between the foot and the sensor position (mid-foot position) decreased. While the second step of the leaning forward posture, the third Flexi force sensor recorded the maximum pressure of 77.82 kPa. Through the analysis of all leaning forward posture data, 77.82 kPa was the maximum pressure established. During the third and fourth steps, the pressure is reduced continuously. Finally, the fourth Flexi force sensor shows a decrease in pressure (almost 0 kPa). Once the subject moves forward to change posture, the heel rises with each step forward. The response of the Flex sensor during the forward-leaning pose is shown in Figure 7. The flexion of the flex sensor, consequently, increases at each step of the leaning forward posture. In the second, third, and fourth steps, the increasing percentage in flexion was 20%, 13%, and 9%, respectively, and 36 Vout was the peak flex sensor response of the leaning forward posture.

**Figure 8:**
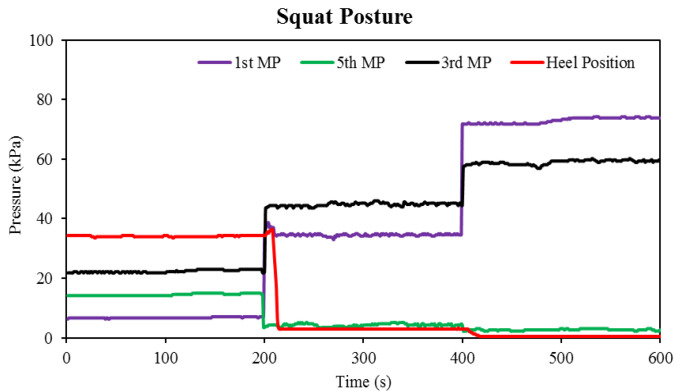
Pressure changes of Flexi force sensors against time during the squat posture.

The change in plantar pressure in the squat position (the third task) is shown in Figure 8. The initial pressures detected at the first, fifth, and third metatarsal and heel positions are 6.85, 14.11, 22.98, and 33.87 kPa, respectively. The first Flexi force sensor positioned on the first metatarsal bone showed an increase in pressure in the semi-squat position. When the subject sat down in the full squat position, the pressure increased by a factor of two. In the third step, the maximum pressure was found because of the flexion of the first foot at this step. Secondly, the subject sits on the foot, so the subject’s entire weight is borne on the forefoot area of the foot, as shown in Figure 4. Compared with the second step (squat position), the foot bending observed in the third step (full squat position) is more pronounced. Among all the postures completed by the subject, the most difficult task is making a half-squat posture because controlling the body balance is a challenge for the subject.

The second Flexi force sensor located on the fifth metatarsal bone shows a drop in pressure during the second and third steps. The results of the first and second Flexi force sensors are opposite to each other. By comparing the results of the first metatarsus with the Flexi force sensor at the third metatarsus, data exhibit alternative pressures during the second and third steps. In the second step of the half-squat posture, the pressure at the third metatarsus showed a 25.64% increase compared to pressure at the first metatarsus. However, in the third step, inverse pressure change was observed at the third metatarsus, showing a 22.55% decrease compared to the pressure at the first metatarsus. The heel Flexi force sensor recorded 2.28 and 0 kPa pressure at the second and third steps, respectively. Figure 7 shows the bend response of the flex sensor while performing a squat posture. During the time of 400–600 s, 39 Vout was the highest bend response obtained by the flex sensor, and in that duration of time, the subject was in a full-squat position. In this figure, it can also be observed that the subject was more stable in a leaning forward posture than in a squat posture because maintaining balance during the half-squat position was a substantial challenge for the subject.

**Figure 9:**
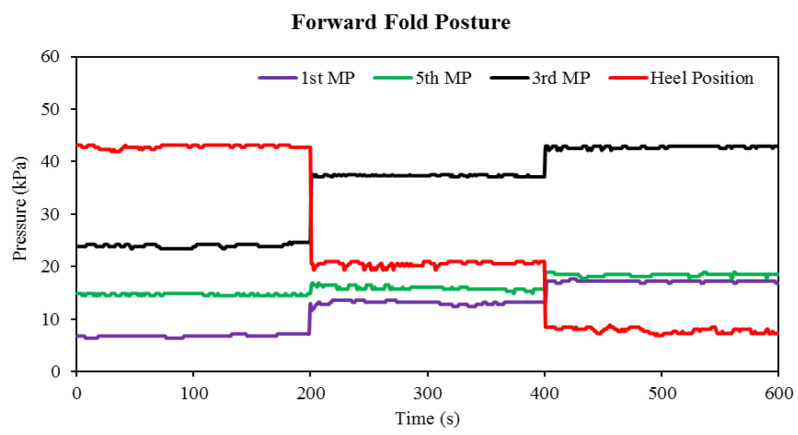
The elapsed time of the pressure change in the Flexi force sensor during the forward folded position.

The results of the forward fold posture (fourth task) are shown in Figure 9. During the standing up straight position (1st step), the pressure values were almost the same as in the case of the squat, leaning forward, and tree postures. The pressure increased on the first metatarsus during the second as well as the third step of the forward fold body posture. Relating to the previous postures, the pressure increment was not as high as in the case of squat posture and leaning forward. The fifth metatarsus positioned Flexi force sensor also showed a minor rise in pressure at the second and third steps because, while performing this posture, only the upper part of the body bent forward, whereas the lower part of the body retained a nearly straight position, as shown in Figure 4. The maximum pressure of the forwarding fold posture was gained at the third metatarsus position during the third step. A 54.19% pressure increase at the second step and 77.34% pressure increase at the third step were obtained compared to the initial pressure values. The response of the heel positioned Flexi force sensor during the forwarding fold posture was different than during the squat and leaning forward postures because, in this posture, the heel of the subject was not wholly raised. In the second and third steps, the pressure decreased 52.38% and 83.3%, respectively, compared to the pressure at the preliminary position.

**Figure 10:**
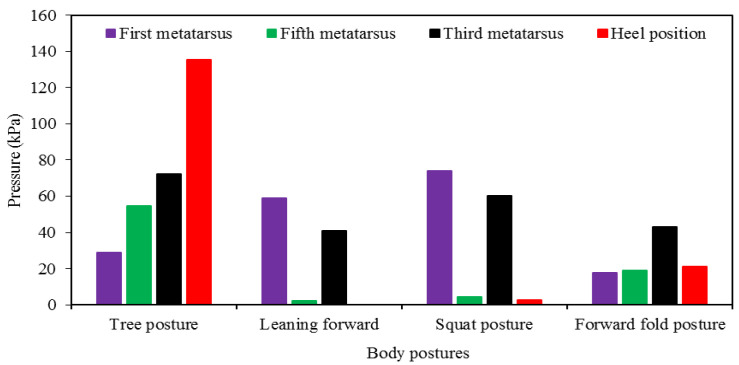
Peak pressure values against body posture at the final step of every posture.

In Figure 10, the highest pressure values of the tree posture, leaning forward, squat posture, and forward fold posture obtained during the last step at the first metatarsus, fifth metatarsus, third metatarsus, and heel position can be shown. The maximum heel pressure recorded during the tree pose was 135.48 kPa; however, the pressure detected during the forward-leaning pose was approximately 0 kPa. At the first metatarsus position, the extreme pressure observed during the squat posture (74.19 kPa) had a 26%, 155%, and 318% pressure increase compared to the leaning forward, tree posture, and forward posture, respectively. By evaluating the peak pressure values of the fifth and third metatarsus positions, it can be determined that the third metatarsus positioned Flexi force sensor showed a 22.6% pressure increase compared to the Flexi force sensor positioned at the fifth metatarsus during tree posture. Leaning forward, the squat posture and forward fold posture also verified that the pressure at the third metatarsus was more than the fifth metatarsus.

**Figure 11:**
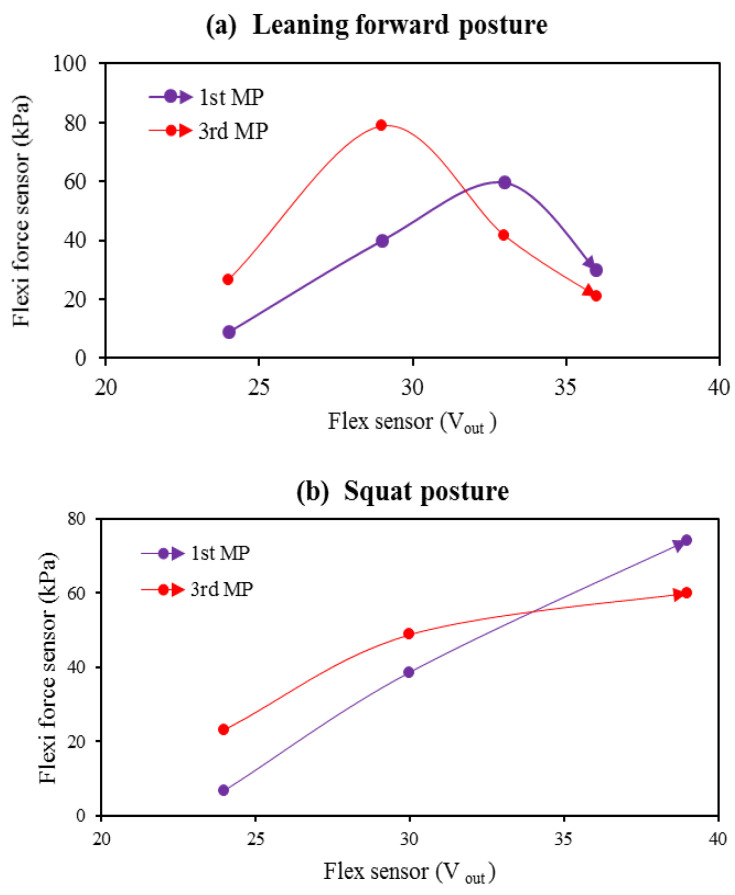
During (**a**,**b**) posture; the Flexi force senor’s response against the flex sensor.

Figure 11 shows the combined results of Flexi force sensors and flex sensor during the leaning forward and squat posture. As mentioned above, the process of bending the foot is only observed during the forward lean and squat postures. When the subject’s foot began to bend, the force was transferred to the forefoot area (1st and 3rd metatarsals). When the subject performed the second step of the forward-leaning posture, the maximum pressure observed at the third metatarsal position was 78.72 kPa.

It was also perceived that during the fourth step, the pressure decreased as the bend angle of the feet increased to their maximum level because, at this stage, the force was transferred from the forefoot area to the toe. During squat posture, the highest pressure was 74.19 kPa recorded at the first metatarsus as the subject bent their feet for a full squat. At the first metatarsus position, the pressure seemed directly proportional to the bend angle, while at the third metatarsus, the pressure was reduced.

**Figure 12:**
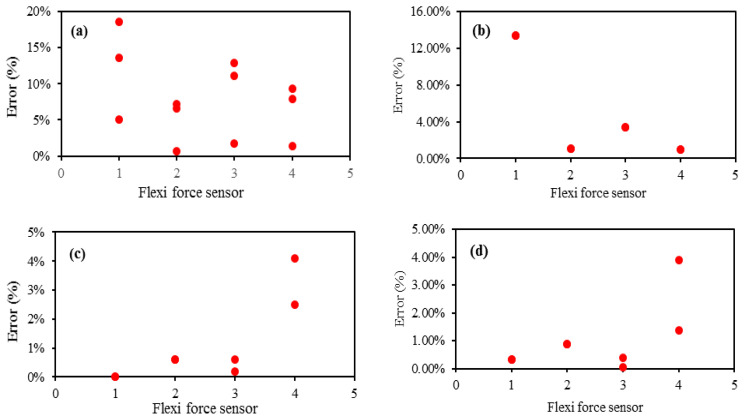
Measured Flexi force sensors error against each sensing position during (**a**) tree posture; (**b**) leaning forward; (**c**) squat; (**d**) forward fold posture.

To study the measurement errors of all Flexi force sensors during each posture performance, the sensing errors that occurred at each step of the postures were calculated by dividing the difference between pressure changes and average pressure. The error percentages at each sensing position during entire postures performed by the male subject are presented in Figure 12. It can be seen that the significant error is 18.6% and was identified at the first metatarsus of the tree posture. Most absolute relative errors were less than 5%. The result of all measured errors demonstrated that the error percentage decreased with the increase in pressure.

## 5. Discussion and Comparison with Previous Related Studies

Insole-based human monitoring systems are valued for their ability to combine different wearable sensors, such as pressure sensors and temperature sensors, and measure body postures that are important in daily life. Although the measurement techniques were found to be similar, the placement of the sensors was found to vary during the manufacturing process [51]. Another smart insole for static posture assessment is proposed. The system consists of a sensor and sensing unit (piezoelectric sensor) with 32 pressure sensor bridges for body monitoring in both motion and static positions [52]. Similar types of smart insoles have also been reported in [53]. IMUs placed on different body parts are used to estimate the pose of the entire body, while smart shoes with air pressure sensors are used to detect movement phases during physical activity. Overall, previous studies and this study have shown satisfactory results for straight walking and full-body movement under static conditions and during motion [54].

## 6. Conclusions

This paper proposes a smart sensing system for measuring plantar pressure distribution under different body postures based on a smart insole based on Flexi force and bending sensors. Together, four Flexi force sensors and a flex sensor were successfully embedded in the insole. Based on a calibration study, measurement studies, typical findings, and conclusions of the four body positions in male subjects are as follows.

(a) The smart insole is designed with wireless Flexi force and flexible sensors to monitor various body positions of a male volunteer subject. The calibration results show that the signal of the linear Flexi force sensor changes with the change in the upper pressure, which proves the reliability and accuracy of the proposed system.

(b) When evaluating the performance of the tree pose Flexi force sensor, the results show that all sensors are well embedded. Therefore, in terms of plantar pressure distribution, various body postures can be sensed using this smart insole. When performing any preparation in any position, when the subject is upright, the maximum pressure occurs at the heel position, and the minimum pressure occurs at the first metatarsal. Therefore, the protection of the heel area is very important.

(c) When the subjects completed the forward, squat, and forward bend positions, the pressure on the first, fifth, and third metatarsals was effectively increased. Combining the above three postures, the extreme pressure of the first metatarsal in the squatting posture increased by 26% and 318% compared with the forward-leaning posture and the forward bending posture, respectively.

(d) The flex sensor response was 18.18% higher in the squat than in the forward lean. The tree pose had the highest error rate of 18.6% for the first metatarsal. 

This smart insole can be used by changing different parameters such as male and female weight, BMI (body mass index), and many other postures. This smart insole can be suitable for sensing various body postures.

## Figures and Tables

**Table 1:** Typical specifications of the Flexi force sensor, flex sensor, PVC, and sports shoe.

(a) Flexi Force Sensor			
Particular	Typical Performance	Evaluation Conditions	
Thickness	0.008″ (0.208 mm)	N/A	
Sensing Area	0.375″ (9.53 mm)	N/A	
Connector	3-pin male square pin	N/A	
Force ranges	Sensor A201-1(4.4 N) Sensor A201-25(110 N) Sensor A201-100(440 N)	N/A	
Linearity	±3% of full scale	Line drawn from 0 to 50% load	
Hysteresis	<4.5% of the full scale	Conditioned sensor, 80% of full force applied	
Repeatability	<±2.5%	Conditioned sensor, 80% of full force applied	
Drift	<5% per logarithmic time scale	Constant load of 111 N (25 lb)	
Response time	<5 µs	Impact load put recorded on an oscilloscope	
Operating temperature	−9 °C to 60 °C	The time required for the sensor to respond to an input force	
**(b) Flex Sensor**			
**Flex Sensor Length**	**Flex Sensor Frequency**	**Maximum Output**	**Measurement Sensitivity**
110 (mm)	30 (Hz)	240 (Vout)	0.56–0.94
